# The impact of the new Dutch guideline on cardiovascular risk management in patients with COPD: a retrospective study

**DOI:** 10.3399/bjgpopen20X101139

**Published:** 2021-01-27

**Authors:** Lonneke ME Nies, Ingrid Looijmans-van den Akker, Liesbeth Rozendaal, Brenda Baar, Rimke C Vos, Huberta E Hart

**Affiliations:** 1 Department of General Practice, Julius Center for Health Sciences and Primary Care, University Medical Center Utrecht, Utrecht University, Utrecht, The Netherlands; 2 Leidsche Rijn Julius Healthcare Centers, Utrecht, The Netherlands; 3 Leiden University Medical Center, Department of Public Health and Primary Care, LUMC-Campus, The Hague, The Netherlands

**Keywords:** risk assessment, risk factors, pulmonary disease, chronic obstructive, cardiovascular diseases, health services research, primary health care

## Abstract

**Background:**

Patients with chronic obstructive pulmonary disease (COPD) have an independent increased risk of cardiovascular (CV) disease. Cardiovascular risk (CVR) assessment should be offered to all patients with COPD, according to the new Dutch CVR management (CVRM) guideline (May 2019).

**Aim:**

To evaluate the impact of the new CVRM guideline on the care of patients with COPD in primary care.

**Design & setting:**

A retrospective study took place within five primary healthcare centres located in The Netherlands.

**Method:**

In accordance with the guideline, the CVR of all patients with COPD was estimated and categorised. Data from 2014–2019 were used for the qualitative risk assessment based on comorbidities, and the quantitative Systematic Coronary Risk Evaluation (SCORE). In addition, the guideline-based follow-up was investigated.

**Results:**

Of the 391 patients with COPD, 84.1% (*n* = 329) had complete data on CVR assessment: 90.3% (*n* = 297) had a (very) high risk, and 9.7% (*n* = 32) a low-to-moderate risk. Of the patients with (very) high risk, 73.4% (*n* = 218) received guideline-based follow-up (primary care: 95.4%, secondary care: 4.6%). In 15.9% (*n* = 62) of all patients with COPD, the CVR profile was not measured and of the (very) high-risk patients, 26.6% (*n* = 79) were not enroled in a CV care programme.

**Conclusion:**

Whereas in the majority of patients with COPD the CVR is already known, for one out of six patients this CVR still has to be assessed according to the recently updated guideline. Moreover, once a (very) high risk has been assessed, as a consequence CV treatment of risk factors should be intensified in one out of four patients with COPD. Adherence to the new CVRM guideline could provide improvement in CVRM in more than a third of all patients with COPD.

## How this fit in

The latest Dutch clinical practice guideline on CVR management, published in May 2019, recommends estimating the risk of CV death in patients with COPD using a qualitative or a quantitative CVR assessment. The aim of this study is to evaluate the impact of this new guideline for the care of patients with COPD in primary care.

## Introduction

Approximately 30–50% of deaths in patients with COPD are attributed to CV events (for example, myocardial infarction).^1–4^ The relative risk of CV morbidity is 2.5 times higher compared with individuals without COPD sharing the same classic CVR factors (for example, smoking)⁠.^5,6^ Whether this relative risk of 2.5 on CV morbidity also holds for CV mortality remains unclear. However, it implies that COPD is an independent predictor for CV disease (CVD). There is increasing evidence that oxidative stress causes pulmonary inflammation and that the remaining circulating pro-inflammatory mediators (cytokines) cause systemic inflammation and induce atherosclerosis.^7^ In addition, the altered mediators of oxidative stress (nitric oxide [NO]) in vascular endothelial cells influence the vessel tone causing vascular dysfunction, a key driver of CVD.^7^

In a population-based cohort study in Dutch community-dwelling middle-aged and older people, an approximately 30% increased risk of sudden cardiac death in those with COPD was found.^2^ The Dutch multidisciplinary guideline on CVRM was used nationally in the last 2 decades to reduce CVD morbidity and death in The Netherlands. Based on aforementioned insight from recent research, the 2019 updated Dutch CVRM guideline advises offering all patients with COPD a CVR assessment.^8,9^ The guideline promotes early treatment for CVR factors in patients with a very high or high CVR by chronic disease management strategies.

Reduction of CVR will require optimal implementation of CVRM.^10–12^ To align the quality improvement of CVRM with the increased importance of CVR assessment every 5 years in all patients with COPD, more insight is needed into whether and how CVR assessment is currently performed in the COPD population. The aim of this study is to evaluate the impact of the new guideline for the care of patients with COPD in primary care. In how many patients with COPD should CVR profiles be measured, and what is the proportion of patients in which treatment should be intensified?

## Method

### Study population and setting

**Figure 1.**
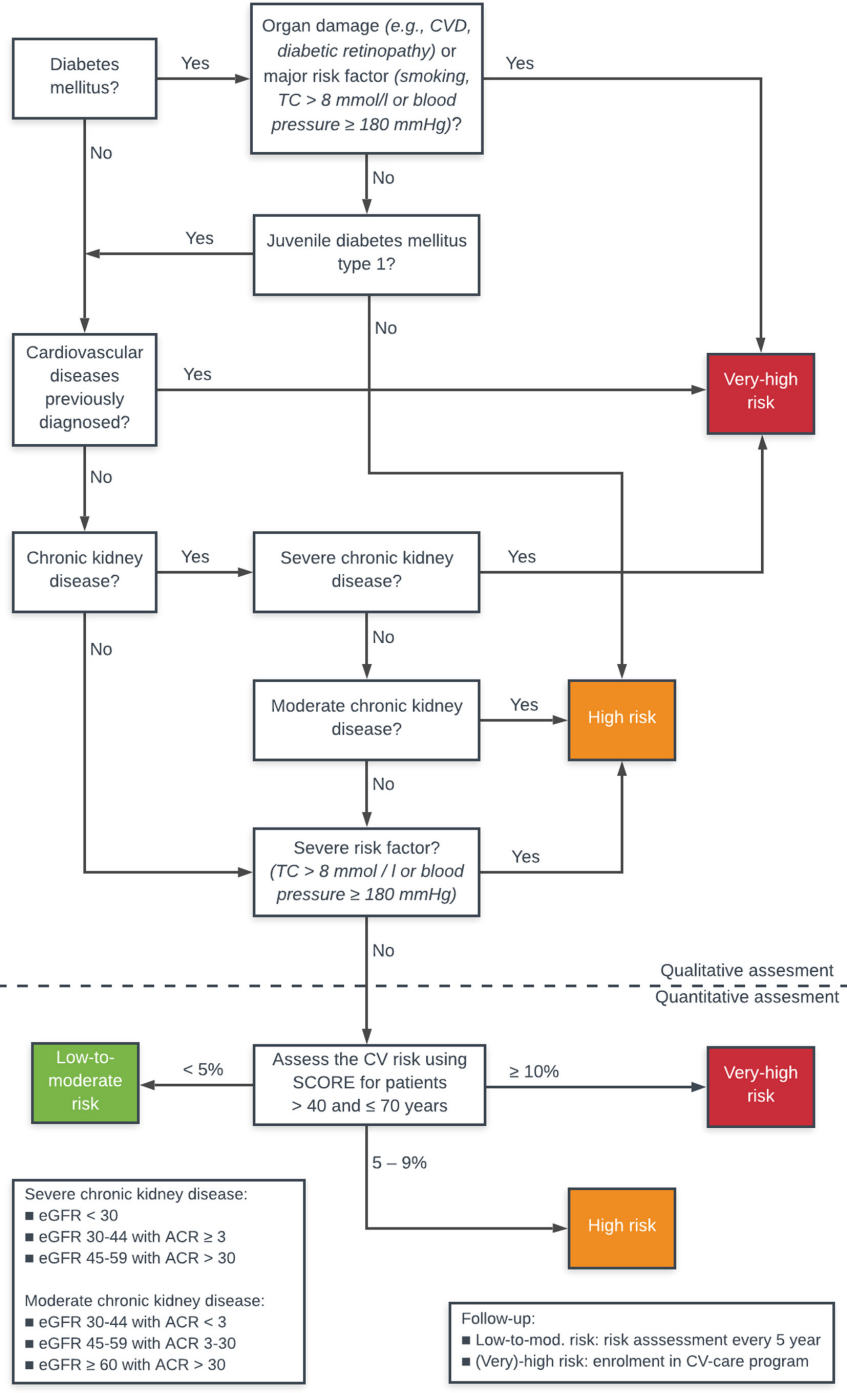
Flowchart risk categories (Dutch CVRM guideline).^8,26^ ACR = albumin-creatinine ratio in urine. CV = cardiovascular. CVD = cardiovascular disease. eGFR = estimated glomerular filtration rate. SCORE = Systematic Coronary Risk Evaluation. TC = total cholesterol.

Within five primary healthcare centres located in the centre of the in The Netherlands, a retrospective study was conducted using routine care data of patients with COPD (International Classification of Primary Care [ICPC] code R95). In accordance with the CVRM guideline, CVR was investigated using the qualitative risk assessment based on CV-related comorbidities, and when absent, the quantitative risk assessment (SCORE) was used (Figure 1).

CVRM is a guideline-based approach to determine and treat all relevant CVR factors to prevent the development of (recurrent) CVD. Patients with any CVR factor have an indication to determine the CVR profile and estimate their CVR by risk assessment. In patients with a low-to-moderate risk, the risk is re-estimated every 5 years. Patients with very high or high CVR are enroled in a CV care programme.

### Data collection

CVR profiles were extracted over 5 years (1 September 2014–31 August 2019), of which most recent measurements in the electronic medical records (EMR) were used. Enrolment rates for the CV care programmes were collected on 31 August 2019. Patients at the healthcare centres had consented to use of their routine care data for scientific purpose and quality improvement evaluation.

#### Patient characteristics

Extracted patient characteristics were sex, age (years), body mass index (BMI, kg/m^2^), Global Initiative for Obstructive Lung Disease (GOLD) classification (I-IV), frailty, and polypharmacy. Data on frailty (proportion of deficits >0.2 by frailty index) and polypharmacy (≥5 chronic medications) were dichotomised (yes or no) and only available for patients aged ≥60 years.^13,14^

#### Cardiovascular risk profiles

Data were extracted on diabetes mellitus (DM), established CVD, chronic kidney disease (CKD), smoking status (current, former, and never), and blood pressure (BP, mmHg). When multiple BP measurements were available, the most recent ones were averaged within a 1-year period. In addition, total cholesterol (TC, mmol/l), low-density lipoprotein cholesterol (LDL-c, mmol/l), TC/high-density lipoprotein cholesterol ratio (TC/HDL-c, ratio), fasting glucose (mmol/l), estimated glomerular filtration rate (eGFR, ml/min/1.73 m^2^), and albumin/creatinine ratio in the urine (ACR-u, mg/mmol) were extracted.

#### Cardiovascular care programmes

Patients with an estimated very high or high CVR have an indication to participate in one of the CV care programmes within chronic disease management. These programmatic consultations provide treatment and follow-up of CVR factors and can be provided in three different CV programmes: ‘diabetes care’, ‘established CVD care’ or ‘(very) high vascular-risk care’. Enrolment status in one of these CV care programmes was extracted from the EMR.

### Cardiovascular risk assessments

After establishing the risk profiles, the CVRs were estimated using a step-by-step hierarchical procedure in accordance with the guideline (Figure 1). In the order detailed below, each patient was assigned to one risk category based on their risk score.

#### Qualitative risk assessment

First, patients with DM were considered to have a ‘very high CVR’, having one of the following complications or risk factors: macrovascular complication (CVD), microvascular complication (nephropathy, neuropathy, retinopathy), positive smoking status, total TC >8 mmol/l, or systolic BP ≥180 mmHg.^15^ Remaining patients with DM were considered to have a ‘high CVR’.^8^

Patients with established CVD (including coronary heart disease, ischaemic cerebral vascular disease, peripheral arterial disease, or atherosclerosis of major arteries) without DM were classified as having a ‘very high CVR’.^8^

CKD was defined as severe or moderate (Figure 1). The remaining patients with severe CKD were considered as ‘very high CVR’ and moderate CKD as ‘high CVR’.^8^

Patients with only one serious CVR factor, such as severe hypercholesterolaemia (TC >8 mmol/l) or severely increased BP (systolic BP ≥180 mmHg), were considered as ‘high CVR’.^8^

#### Quantitative risk assessment

For patients who had no data on qualitative CVR assessment, the absolute 10-year risk of fatal CVD event was estimated using the SCORE algorithm based on sex, age, smoking status, BP, and TC/HDL-c.^8^ The guideline describes that regardless of risk factors, patients aged <40 years do almost all have an absolute ‘low-to-moderate CVR’, and patients aged >70 years an absolute ‘very high CVR’. Those patients were classified as such when having complete data regarding the SCORE.

For patients using antihypertensive therapy and/or lipid-lowering therapy, the risk could not be examined by SCORE owing to the use of CV medication.^8^ It was assumed that these medications were initiated by a quantitative estimated (very) high CVR. Those patients were considered as having an absolute ‘high CVR’, regardless of completeness of data.

#### Cardiovascular risk categories

Patients were classified as having a ‘very high’ (≥10%), a ‘high’ (≥5% and<10%) and a ‘low-to-moderate CVR’ (<5%). For patients with incomplete data on both assessments, CVR could not be assessed. They were considered to have an ‘unknown CVR’, and only the patient characteristics were described and the risk factors reported.

### Analysis

Descriptive statistics were used. Data are shown as numbers with percentages, or means with standard deviation (SD). Median with interquartile range (IQR) was used when variables were not normally distributed. Statistical analyses were performed using SPSS statistics (version 25.0).

## Results

### Study population

**Figure 2.**
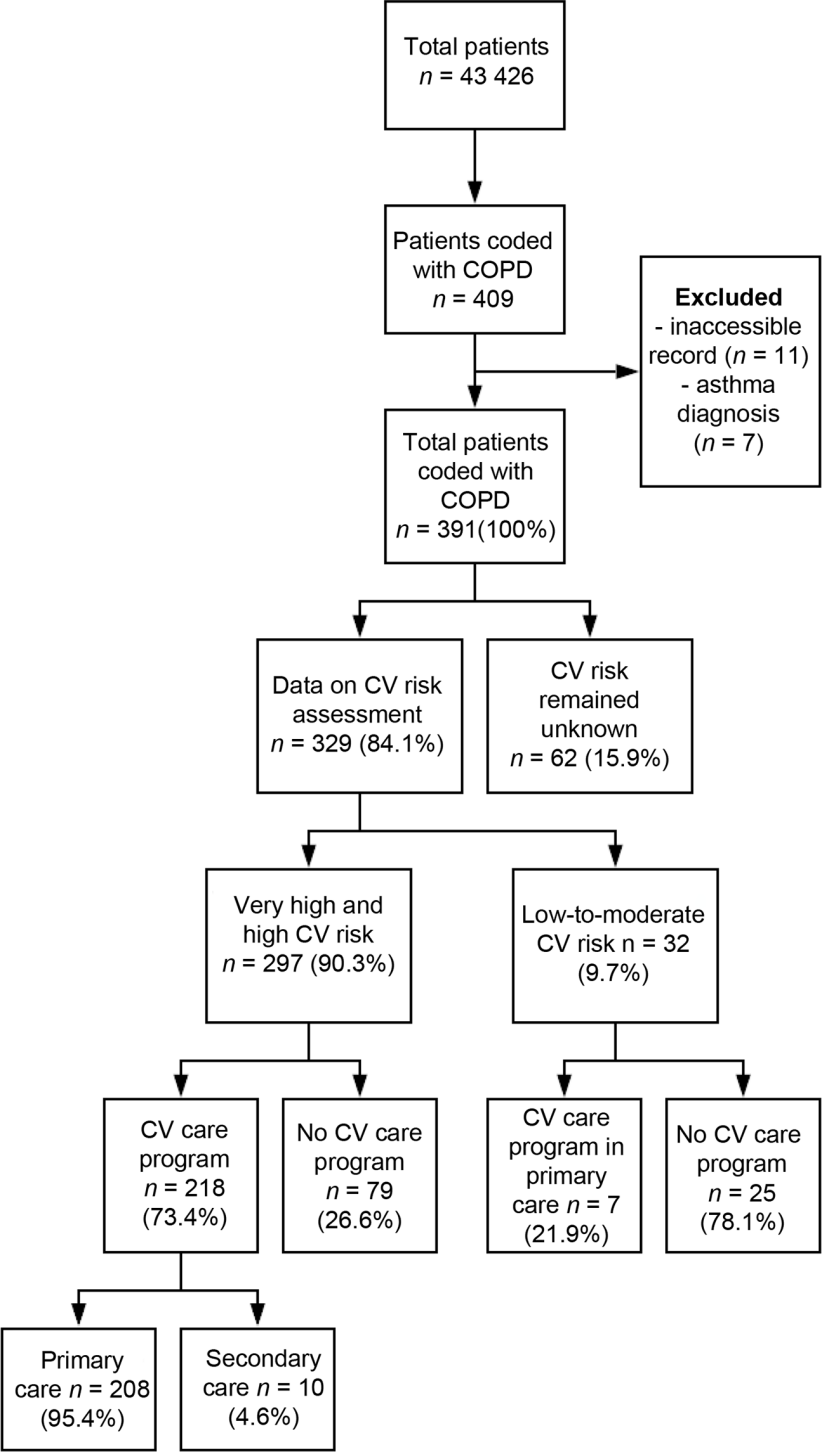
Flowchart: cardiovascular risks and (un)provided cardiovascular care programme. CV = cardiovascular

There were 43 426 patients registered within the healthcare centres, of whom 391 were diagnosed with COPD, which is a 0.9% prevalence of COPD (Figure 2).

The patient characteristics are shown in Table 1. Mean age was 67.5 years (SD =10.8) and 46.5% were male. A total of 43.5% of the patients were currently smokers. Three out of four of the COPD population were aged ≥60 years; of those older patients, 64.0% were frail and 56.5% used polypharmacy.

**Table 1.** Patients characteristics (*n* = 391)

	***n***	***%***
**Male**	182	46.5
**Mean age**, years ± SD<4040–70>70	67.5 ± 10.81235155	-0.360.139.6
**Smoking status**		
CurrentNeverFormer*Missing*	17025173*23*	43.56.444.2*5.9*
**Median** **BMI**, kg/m^2^ (IQR)*Missing*	26.4 (7.2)*70*	*-* *17.9*
**COPD classification**		
GOLD 1GOLD 2GOLD 3GOLD 4*Missing*	103189648*27*	26.348.316.42.0*6.9*
**C** **haracteristics** **of patients aged** **≥** **60** **years** **(** ***n*** **=** **294** **)**		
**Frailty** ^a^		
YesNo*Missing*	18849*57*	64.016.7*19.4*
**Polypharmacy** ^b^		
YesNo*Missing*	16671*57*	56.524.1*19.4*

^a^Proportion of deficits >0,2 by frailty index. ^b^Five or more chronic medications. BMI = body mass index. SD = standard deviation. IQR = interquartile range.

### Cardiovascular risk categories

The CVR categories were based on the estimated CVRs by qualitative and quantitative risk assessments. Data on risk assessment were complete in 329 of the 391 patients with COPD (84.1%) and revealed 297 (very) high-risk patients (90.3%) and 32 low-to-moderate-risk patients (9.7%) (supplementary Table 1). The CVR remained unknown for 62 patients with COPD (15.9%). Of the patients with (very) high risks, 178 had a very high CVR (59.9%) and 119 a high CVR (40.1%). By far the largest risk groups were 'DM' (*n* = 87), 'CVD without DM' (*n* = 84), and 'CV medication without qualitative risk' (*n* = 85). The estimated risks included 183 qualitative (55.6%) and 146 quantitative (44.4%) risks.

#### Unknown cardiovascular risk group

Of the patients with unknown risks, 48.4% were current smokers. Almost half of the patients were aged ≥60 years and, of those, 41.4% were frail and 24.1% used polypharmacy. The lipid spectrum was missing in 93.5%, systolic BP in 25.8%, and smoking status in 16.1% (supplementary Table 2).

### Cardiovascular care programme

Of the 297 (very) high-risk patients, 218 patients (73.4%) received guideline-based follow-up: 208 (95.4%) a CV care programme in primary care and 10 (4.6%) in secondary care. The remaining 79 patients (26.6%) was not enroled in a CV care programme, as they should be (Figures 2 and 1).

#### Unprovided cardiovascular care programme

**Figure 3.**
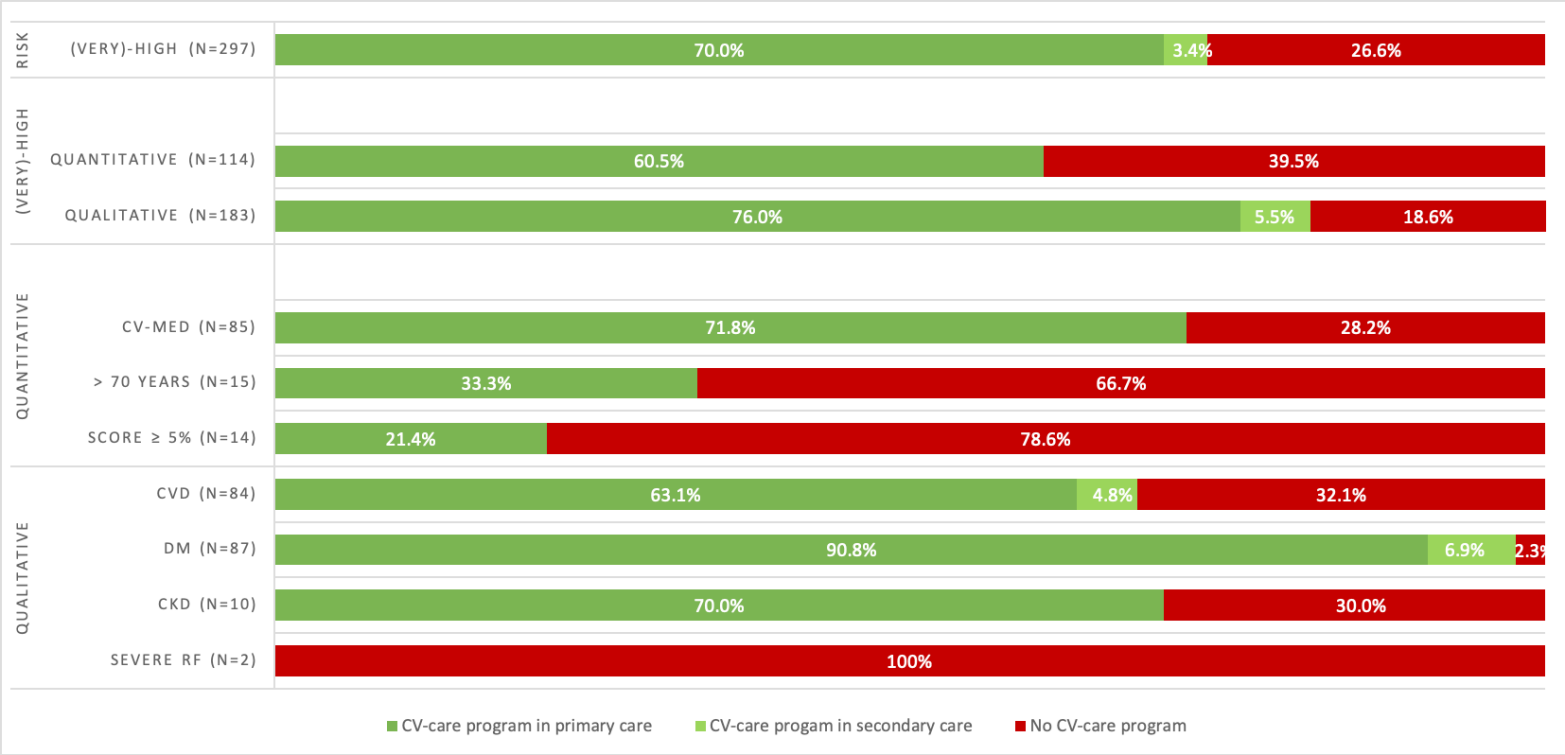
Cardiovascular risk and participation in cardiovascular-care programme of patients with COPD. CKD = chronic kidney disease. CV-med = cardiovascular medication. CVD = cardiovascular diseases. DM = diabetes mellitus. RF = risk factors. SCORE = Systematic Coronary Risk Evaluation.

The proportion of ‘(very) high-risk patients without CV care programme’ was two times higher for the quantitative risk group than for the qualitative risk group (*n* = 34, 39.5% versus *n* = 45, 18.6%) (Figure 3). Almost all patients with DM (97.7%) received programmatic CV care, while 32.1% of the patients with CVD did not receive this care (Figure 3). A difference in percentage of ‘unprovided CV care programme’ was found within the very high- and high-risk categories (23.6% versus 31.1%; supplementary Table 1).

## Discussion

### Summary

This study aimed to evaluate the impact of the new Dutch CVRM guideline, including the CVR assessment of patients with COPD and their consequent indication for follow-up. Data on qualitative or quantitative CVR assessment was already complete in almost 85% of all patients with COPD, despite the fact that the new guideline had only just been published and was, therefore, not yet fully implemented. Of the patients with complete data, 90.3% had a very high or high CVR; of them, 73.4% received a CV care programme for their (very) high CVR, mostly in the primary care setting (95.4%). The results show that, currently in 36.1% (*n* = 141) of all patients with COPD, there is room for improvement in CVRM; namely by completing the CVR profiles of the patients with unknown CVRs (*n* = 62) or by enroling (very) high-risk patients in the CV care programme of chronic disease management they need (*n* = 79). The results may help to draw clinical attention to patients with COPD, especially to those with (potential) quantitatively high or very high CVRs.

### Strengths and limitations

This study describes the assessment of CVRs and care provided for (very) high CVR in a Dutch cohort of patients with COPD. It underlines the urgency of assessment of CVR in all patients with COPD, to reduce the incidence of CVD in these patients over the next decades.

Making use of reported risk factors for CVR assessment, the study identified all patients with COPD with an ‘unknown’ or a ‘possibly known’ CVR within this general practice. CVRs were determined by extracting data from the EMR, but it is not known whether the healthcare professional actually combined the various data to determine the CVR. This could have caused an underestimation of the ‘unknown CVRs’. Therefore, the authors have tried to identify this underestimation by determining the ‘(very) high-risk patients not enroled in a CV care programme’, given that these patients already had an indication and should be enroled. By combining the ‘unknown risks’ and the ‘(very) high risks without CV care programme’, a good estimation could be made of the possible impact of the new guideline by adherence.

A clear limitation in this study is that some assumptions were made when the SCORE was not applicable. Since CV medication use is an indication for enrolment in a CV care programme, it was assumed that patients who use CV medication had a ‘high CVR’. However, it was not known for certain whether the healthcare professional determined this risk before starting drug therapy. In accordance with the guideline, it has also been assumed that patients aged <40 years had a ‘low-to-moderate CVR’ and that patients aged >70 years had a ‘very high CVR’ using the SCORE.

### Comparison with existing literature

#### The impact of the new guideline (unknown risks)

According to the new Dutch guideline, the CVR should be known for every patient with COPD.^3^ By estimating CVRs with reported risk factors, it has been shown that 15.9% (*n* = 62) of patients had incomplete data on CVR assessment. This was mainly owing to an unknown lipid spectrum. As expected, smoking status was well recorded, being a disease variable that has traditionally been a part of COPD care. This finding underlines the importance of a structured approach for recording CVR profiles into COPD disease management programmes.

The primary care centres where the study was conducted are located in a newly developed large residential area near Utrecht. The average age in this area is slightly younger than the overall Dutch population (32.1 years versus 41.0 years). Since the onset of COPD exceeds middle age, the prevalence in the study population is lower than nationwide prevalence (0.9% versus 1.9%). This could very well mean that, nationwide, an even larger number of patients with COPD will still need CVR assessment.^16,17^

Compared with the total COPD population, the unknown risk group included more younger patients and less polypharmacy, but more active smokers. Even though it is likely that younger patients with COPD have a lower absolute risk of CVD, it is still important to estimate their risk. Relatively speaking (individuals with COPD versus without COPD): the younger the patient, the higher the relative CVR, regardless of sex.^13,18,19^ Therefore, the largest disease gain can be achieved for smokers starting with a guideline-based approach of risk assessment and counselling at a young age.

#### Guideline adherence (CV care programme)

In the study, 90.8% of the 87 patients with COPD with DM did receive the CV care programme in primary care. Contrastingly, only 63.1% of the 84 patients with COPD with CVD (without DM) received this programme. A hypothesis for the difference in care provided could be that CV care for patients with DM can be standardised more easily than the diverse CVD group, which includes different diagnoses with different medical specialists. The Dutch 2018 report on programmatic CV care for general patients also revealed a 20% difference between CVD (without DM) and DM (65% versus 85% in first line).^17^ They argue for better cooperation: the specialist should refer the patient back to primary care if only CVRM is required, and primary care should remain attentive to this.

Furthermore, progress can be made in the provision of CV care to patients with a quantitative (very) high CVR, with greater awareness of reported CVR factors in patients who do not yet have CV-related comorbidity. Only 60.5% of the patients with COPD with a quantitative (very) high CRV, received the CV care programme as indicated. The proportion of ‘(very) high-risk patients without CV care programme’ in the quantitative group was twice as high as in the qualitative group. Similar results were found by Ludt *et al* over the period 2008–2009; they advocated the use of risk assessment and a focus on individuals at risk before CVD is diagnosed.^20^

### Implications for practice

Consistent with the literature, the study underscores the common prevalence of CV-related comorbidities in this CV at-risk population.^21,22^ Patients with COPD often had multimorbidity, which is frequently overlooked when diagnosing an initial chronic disease.^23^ This is especially important in diseases such as COPD, in which treatment is purely symptomatic and does not address underlying pathophysiological causes. Therefore, there is a need to proactively start screening on CVR factors in patients with COPD.^24,25^

In conclusion, the implementation of effectively recording CVR factors and assessing CVRs in primary care is an important first step in the prevention of CVD in patients with COPD. In the majority of patients the CVR is already known, but for one out of six patients with COPD this risk still has to be examined, according to the recent CVRM guideline. Moreover, once a (very) high risk has been estimated, as a consequence, treatment of risk factors should be intensified in one out of four patients with COPD with a (very) high risk. Certainly, in patients with quantitative (very) high CVRs, additional attention should be paid to ensuring appropriate follow-up of risk factors. Adherence to the new CVRM guideline by healthcare professionals could provide improvement in CVRM in more than a third of patients with COPD.
